# Relationship between Pain Intensity, Physical Factors, Pronociceptive Pain Modulation Profile and Psychological Vulnerability on Upper Limb Disability in Older Patients with Chronic Shoulder Pain

**DOI:** 10.3390/ijerph192215006

**Published:** 2022-11-15

**Authors:** Geraldine Valenza-Peña, Javier Martín-Núñez, Alejandro Heredia-Ciuró, María Granados-Santiago, Laura López-López, Marie Carmen Valenza, Irene Cabrera-Martos

**Affiliations:** 1 Department of Physiotherapy, Faculty of Health Sciences, University of Granada, 18016 Granada, Spain; 2 Department of Nursing, Faculty of Health Sciences, University of Granada, 18001 Granada, Spain

**Keywords:** disability, fear-avoidance beliefs, shoulder pain, physical variables

## Abstract

Background: Chronic shoulder pain is a very prevalent condition causing disability and functional impairment. The purpose of the study was to evaluate the relationship between pain intensity, physical variables, psychological vulnerability, pronociceptive pain modulation profile and disability in older people with chronic shoulder pain. Methods: A cross-sectional study was carried out. A total of 56 participants with non-specific chronic shoulder pain of the “Complejo Hospitalario Universitario” (Granada) and 56 healthy controls were included. The outcomes evaluated were pain intensity (visual analogue scale), physical factors (dynamometry for grip strength), psychological vulnerability (Fear-Avoidance Beliefs Questionnaire and Tampa Scale of Kinesiophobia), pronociceptive pain modulation profile (pain pressure algometry) and disability (Quick Disability Arm Shoulder Hand questionnaire). Results: Disability showed a positive correlation with pain and psychological vulnerability (*p* < 0.05) and a negative correlation with pronociceptive pain variables and dynamometry (*p* < 0.001). Psychological vulnerability also presented a strong negative correlation with proprioceptive pain variables and dynamometry and a positive correlation with pain (*p* < 0.05). In regard to the pronociceptive pain modulation profile, a strong negative correlation with pain (*p* < 0.001) and a positive moderate correlation with dynamometry (*p* < 0.001) were shown. Conclusions: Our results support a strong association between disability, psychological vulnerability and pronociceptive pain modulation profile in older adults with chronic shoulder pain.

## 1. Introduction

Shoulder pain is the third most frequent reason why people seek treatment from a healthcare professional [1], after back and neck pain [2], being responsible for enormous health care costs [3]. Chronic shoulder pain tends to increase with age and be more prevalent in women, in persons from lower socioeconomic groups and in psychologically stressed populations [4]. Older people have a high prevalence of non-specific shoulder pain (31% with severe pain intensity), and they present associated functional limitations, which are often under-treated and could affect their quality of life [5]. While there is evidence that suggests conservative interventions can reduce pain and improve function, 50% of patients report persistent pain at six months, and 40% still have pain at one year [6,7].

Objective medical assessment, including tissue pathology, have been found to not be correlated with the severity and chronicity of shoulder pain in older adults [5,8], suggesting that the underlying pathophysiology may be more complex due the age-associated comorbidities. The experience of pain is sculpted by a mosaic of factors unique to the person, including demographic variables, genetic factors and psychological processes, which renders the pain experience completely individualized. Previous studies have shown that patients with chronic pain pathologies could present a dysfunction of the nociceptive system [9,10] or a psychological susceptibility which can contribute to developing and maintaining this pain condition [11,12]. Moreover, strong associations between psychosocial factors and the experience of pain have been reported, with increased psychological distress, greater life stress, and more non-pain somatic symptoms in people with chronic pain conditions.

Shoulder pain patients often are not able to perform certain activities because they anticipate that such activities will increase pain and suffering [13]. Avoidance behavior results in the reduction in both social and physical activities (loss of mobility, muscle strength and fitness) which leads to a number of physical and psychological consequences (loss of self-esteem, deprivation of reinforces, depression, somatic preoccupation), increasing the disability [14]. This vicious cycle directly interferes in the person’s recovery, which reduces treatment adherence and preserves this negative pain experience. In this line, psychological processes have been proposed as mediators in the pain experience becoming crucial in the development of chronic musculoskeletal pain.

The older population has proven difficulties in the identification of physical factors related to shoulder pain [5]; furthermore, little is known about the nociceptive process and/or psychological vulnerability in a frequently disabled population. So, the aim of this study is to introduce an analysis between physical factors, pronociceptive variables, psychological vulnerability and disability in older patients with chronic shoulder pain.

## 2. Methods

An observational cross-sectional study was carried out. Patients diagnosed with non-specific chronic shoulder pain were recruited from the Rehabilitation Service of the “Complejo Hospitalario Universitario” (Granada), between September and April 2019. They had to be aged 65 or over and suffering from non-specific chronic shoulder pain, with symptom duration of more than three months [1]. Exclusion criteria were inability to complete the questionnaires and the presence of significant upper extremity motor comorbidities, as a concomitant neurological condition.

Participants were informed about the purpose and procedures of this study and gave their written informed consent prior to their involvement. A control group, which consisted of age and sex-matched healthy volunteers who had no chronic shoulder pain, was included. The study protocol was approved by the Human Research Ethics Committee of the Hospital. The procedures followed were in accordance with the ethical standards of the responsible committee on human experimentation and with the Helsinki Declaration of 1975 [15].

### 2.1. Measurements and Procedures

A normalized interview and an initial assessment were carried out when inclusion criteria were confirmed. Sociodemographic data and pain characteristics included age, sex, body mass index (BMI) occupation, pain duration and the initial side of pain.

Main outcomes included physical factors, psychological vulnerability, pronociceptive pain modulation profile and disability.

#### 2.1.1. Pain Intensity

Pain severity was assessed using the Visual Analogue Scale (VAS). The VAS is commonly used to assess the subjective aspects of pain from 0 to 10, where 0 reflects no pain and 10 reflects the worst possible pain. Participants were asked to mark a spot on a line of 10 cm from 0 to 10 indicating their average experience of pain during last week.

#### 2.1.2. Physical Factors

Grip strength was measured using a Jamar dynamometer (Promedics, UK) with a standard protocol allowing three attempts on each side [16]. During each measurement, patients were seated with their shoulder adducted and their elbow flexed to 90°. The highest value reached was used in the statistical analyses. The Jamar dynamometer is the most commonly used tool for measuring grip strength with low reported between-observer variability [17] and good test–retest reliability [18]. It has been previously reported that the handgrip activity is an important measure to include in the clinical assessments of patients with shoulder pain [19].

#### 2.1.3. Psychological Vulnerability

Kinesiophobia and fear-avoidance beliefs [20] were included:

The Tampa Scale of Kinesiophobia (TSK) is used to assess fear of (re)injury due to movement. It has 17 items, scored on a 4-point range. Patients are asked to rate their degree of agreement with each of the 17 statements. Four of the items are reverse scored. Scores range between 17 and 68, with higher values reflecting greater fear of (re)injury. Its reliability and validity have been previously reported.

The Fear-Avoidance Beliefs Questionnaire (FABQ) was developed to measure patients’ beliefs about how physical activity and work affect their pain. The self-reported questionnaire consists of 16 independent sentences that are rated by participants on a 7-point Likert scale from 0 (completely disagree) to 6 (completely agree). The questionnaire is divided into two parts. The FABQ–Physical Activity subscale measures attitudes and beliefs about physical activities. The FABQ–Work subscale assesses participants’ attitudes and beliefs about how occupational activities may influence pain. In both subscales, a high score indicates strong fear-avoidance beliefs. This questionnaire has been validated in the Spanish language [21].

#### 2.1.4. Pronociceptive Pain Modulation Profile

The pressure pain threshold was measured using a pressure algometer (Somedic AB, Farsta, Sweden). This device consists of a round rubber disk (1 cm^2^) attached to a pressure (force) gauge (kg) ranging from 0 to 10 kg (kg/cm^2^). The pressure was applied approximately at a rate of 1 kg/cm^2^/s, with the algometer placed perpendicular to the application point. All measures of PPT were administered by the same researcher. Subjects were informed that the research aimed at determining the individual pain threshold, not pain tolerance. The participants were instructed to say stop when they feel they reach their pain threshold. PPT levels were assessed over the 2 points previously identified and marked located bilaterally over the anterior aspect of the shoulder and trapezium [22]. The PPT for each point was measured three times in order to obtain an average value. A 30 s resting period was allowed between each trial. PPT has been shown to be a highly reliable measure to evaluate pain [23] and it has been previously used in patients with chronic pain [24].

#### 2.1.5. Disability

Quick Disability Arm Shoulder Hand questionnaire (QuickDASH) [25]: The QuickDASH questionnaire is a shortened version of the DASH Outcome Measure [26]. Instead of 30 items, the QuickDASH uses 11. It measures the symptoms as well as the ability to perform certain activities referring to the past week. The quick version of the questionnaire has been found to maintain an acceptable internal consistency for individual patient evaluation [27] and a good test–retest reliability and responsiveness in patients with shoulder pain [25]. Raw scores are converted to a 0–100 continuum score. A higher score indicates greater disability. The Spanish version of the QuickDASH was used in this study (available in http://dash.iwh.on.ca/, accessed on 20 April 2020).

### 2.2. Data Analysis

Data were analyzed using IBM SPSS version 20.0. Prior to statistical analysis, the Kolmogorov–Smirnov test was performed to assess the normality. All numerical variables were expressed as the mean ± standard deviation (SD). The t-student test was used to calculate the between groups differences. A 95% confidence interval was used for statistical analysis. A bivariate correlation analysis was conducted between pronociceptive variables, psychological variables and disability scores. Coefficient of Spearman’s rho was used to determine the association between the physical, psychological and disability measures. The strength of the correlations was based on the criteria described by Portney and Watkins [28]: values < 0.25 indicate little or no relationship; 0.25–0.50 suggest a fair relationship; 0.50–0.75 represent a moderate to good relationship, and values above 0.75 suggest an excellent relationship. A *p* value <0.05 was considered statistically significant.

## 3. Results

**Figure 1:**
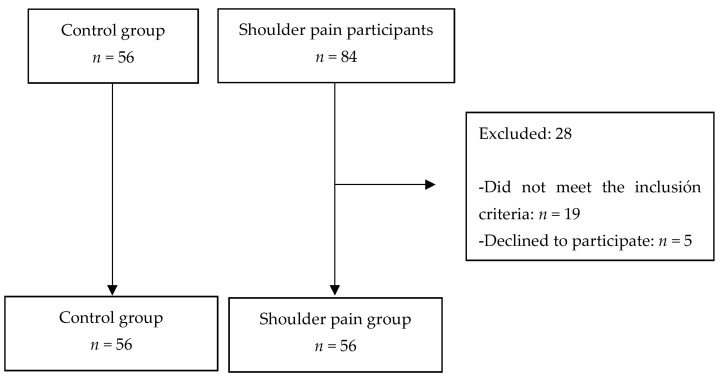
Flow chart.

A total of 84 participants were initially screened for eligibility. Twenty-eight patients were excluded because they did not meet the inclusion criteria (ten were under 18 years old, thirteen participants suffered from less than three months of pain, and five patients had comorbidities that affect the execution of the proposed tests). The final sample was composed of 56 people with shoulder chronic pain. The distribution of the participants is shown in Figure 1.

Demographic characteristics of the participants and pain characteristics are shown in Table 1.

No significant differences were found between groups in the percentage of men and women (61.11% vs. 35.29%), the BMI (*p* = 0.452) or the occupation (*p* = 0.127). The mean age presented significant differences between groups (*p* = 0.001), with older participants in the control group. In regard to pain characteristics, the duration of pain was around 3 years, and the right arm was the most prevalent in the start (76.47%).

Differences between groups in physical factors, psychological vulnerability, pronociceptive pain modulation profile and disability are presented in Table 2.

Significant differences were found between groups in right shoulder pain (*p* = 0.003) and left shoulder pain (*p* = 0.006); however, the handgrip strength did not present significant differences in none of the sides (*p* > 0.05), although the shoulder pain group presented lower scores. In regard to pain pronociceptive profile, the control group presented significant greater thresholds in all measured points compared to the painful group (*p* < 0.05). The psychological vulnerability presented significant differences between groups. The shoulder pain group presented high psychological vulnerability, with poorer scores in the FABQ1 (*p* = 0.036), FABQ total (*p* < 0.001) and TSK (*p* < 0.001). The QuickDASH also presented significant differences between groups, with a higher disability in the shoulder pain group (*p* < 0.001).

Correlations between physical factors, psychological measures, pronociceptive pain variables and disability are presented in Table 3.

Disability showed a strong positive correlation with right shoulder pain (r = 0.779, *p* < 0.001) and a fair positive correlation with left pain (r = 0.396, *p* < 0.05). A significant fair negative correlation was found between disability and both sides dynamometry and a strong negative correlation with the pronociceptive pain variables (*p* < 0.001). Participants with more disability also showed a strong correlation with more fear-avoidance beliefs and kinesiophobia (*p* < 0.001). Psychological vulnerability presented a strong negative correlation with most proprioceptive pain variables, and participants with poorer pain thresholds presented greater fear-avoidance beliefs and kinesiophobia. Moreover, psychological vulnerability presented a moderate positive correlation with pain (*p* < 0.05) and a negative correlation with dynamometry, and participants with poorer strength presented more kinesiophobia (*p* < 0.05). In regard to the pronociceptive pain profile, a strong negative correlation with pain (*p* < 0.001) and a positive moderate correlation with dynamometry (*p* < 0.001) were shown, and patients with more pain and lower strength presented a greater pronociceptive pain modulation profile.

## 4. Discussion

The purpose of the study was to evaluate the relationship between physical and psychological variables, pronociceptive pain profile and disability in participants with chronic shoulder pain. Disability showed a strong positive correlation with pain, psychological vulnerability and pronociceptive pain variables, and a fair negative correlation with dynamometry. Moreover, psychological vulnerability presents a significant correlation with pain, strength and pronociceptive pain variables.

The sample size included in our study and the clinical profile of the participants are similar to other studies focused on shoulder pathology [29,30,31].

Disability reported a significant correlation with physical and psychological variables. Regarding this finding, a more specific approach should be needed including a physical and a psychological assessment in participants with chronic shoulder pain. A relationship between chronic pain and disability has been previously found in chronic conditions such as fibromyalgia [32], low back pain [33,34] or whiplash-associated disorders [35]. Keefe et al. [36] suggested that patient’ beliefs about the cause and treatment of pain may mediate changes in physical disability following participation in a multidisciplinary pain management program, not only physical measures which are clearly related to chronic musculoskeletal pain [37,38].

Previous studies have found that the evaluation of fear-avoidance response is important because it generates a predictive profile of disability and subjective pain [33]. Crombez et al. [39] suggested that pain-related fear is more disabling that pain itself. In fact, personal psychological factors may contribute to the development and increase in disability in musculoskeletal pain. Phobia to movement and behavior of movement avoidance caused by fear are related (*p* < 0.001) to the pain pressure threshold, grip strength and self-reported pain values in patients with chronic shoulder pain in our study. The findings obtained are consistent with those previously reported by other authors [40,41]. A high percentage of people fear movement, and it causes pain and a negative impact on physical and clinical variables, pain intensity and disability. These nocebo effects are known to possibly play a role in the transition from acute pain into chronic pain [42].

Finally, our results show an important correlation between psychological vulnerability and pronociceptive pain modulation profile. Participants with more fear-avoidance beliefs and kinesiophobia present a sensitization of the central neural system, with an amplified response and/or increased responsivity of nociceptive neurons. Previous studies have suggested that psychological factors could contribute to a pronociceptive pain profile creating a vicious circle that could be keeping the pain [43,44]. The reduction in activity due to these wrong thoughts may serve to sustain or exacerbate chronic pain, contrary to the individual’s intent, and may result in even more activity restriction and subsequently, functional limitations due to physical deconditioning [44].

It is important to consider these findings in a clinical context, improving aspects which involve the approach of patients with chronic shoulder pain. Our results are consistent with the hypothesis that not only a physical approach is needed to explain results in negative therapeutic responses. Therefore, it is important to know the specific profile of chronic shoulder pain patients and to take into account fear-avoidance situations in order to adapt the rehabilitation program to them. Domenech et al. [45] also reported that it is possible to change individuals’ behavior focusing on beliefs and attitudes instead of conducting an excessively medical intervention.

Several limitations of this study should be noted. First, it had a small sample size; larger sample sizes could improve the results reliability. However, previous studies in this population had used similar sample sizes [29,46]. Secondly, there was a lack of a follow-up to determine if the relationship is maintained along the time. Nevertheless, similar studies in this pathology did not include one [47].

Future studies should examine the effectiveness of specifics programs, considering psychological aspects of fear-avoidance and kinesiophobia and the pronociceptive pain modulation profile in the assessment of patients with chronic shoulder pain. Thus, our findings could help to improve the approach of older people with chronic shoulder pain, improving their functionality and quality of life.

## 5. Conclusions

Our study shows important associations between disability, psychological vulnerability and pronociceptive pain variables in patients with chronic shoulder pain. Fear of movement/(re)injury should be taken into account because it could influence the perception of disability and the central nervous system sensitization. So, the goal of future research is to deploy personalized shoulder pain management, developing not simply a treatment based on biological profile, but rather a truly personalized therapy based on a multidisciplinary treatment.

## Figures and Tables

**Table 1:** Demographic and pain characteristics of the participants.

	Shoulder Pain Group (*n* = 56)	Control Group (*n* = 56)	*p*
Sex (% men)	61.1	35.2	0.127
Age (years)	54.35 ± 9.23	66.94 ± 11.50	0.001 *
BMI (kg/m^2^)	26.09 ± 3.08	25.24 ± 3.51	0.452
Occupation (%)			
Physical jobs Not physical jobs	61.1 38.8	35.2 64.7	0.127
Pain duration	3.39 ± 0.92	-	-
Initial side of pain (% right)	76.4	-	-

BMI: body mass index; kg: kilograms; m: meters. Variables are expressed as mean ± standard deviation or percentage (%). * *p* < 0.05.

**Table 2:** Differences between groups in physical factors, pronociceptive pain profile, psychological vulnerability and disability.

	Shoulder Pain Group (*n* = 56)	Control Group (*n* = 56)	*p*
Pain intensity			
VAS right	4.36 ± 2.26	1.9 ± 2	0.003 *
VAS left	3.39 ± 3.67	0.53 ± 1.33	0.006 *
	Physical factors		
Dynamometry right	25.4 ± 9.29	29.95 ± 12.98	0.240
Dynamometry left	24.94 ± 8.29	29.87 ± 13.41	0.197
Pronociceptive pain modulation profile			
Shoulder algometry right	2.54 ± 1.04	4.71 ± 1.67	<0.001 **
Shoulder algometry left	2.68 ± 1.35	4.85 ± 1.99	0.001 *
Trapezium algometry right	3.63 ± 1.96	5.13 ± 1.99	0.032 *
Trapezium algometry left	3.49 ± 1.68	5.55 ± 1.73	0.001 *
Psychological vulnerability			
FABQ 1	15.56 ± 7.22	10.76 ± 8.59	0.083
FABQ 2	17.44 ± 11.61	9 ± 11.27	0.036 *
FABQ total	33 ± 17.87	19.29 ± 14.23	<0.001 **
TSK	45.94 ± 7.5	32.53 ± 11.98	<0.001 **
Disability			
QuickDASH	42.8 ± 20.09	18.24 ± 16.83	<0.001 **

VAS: visual analogue scale, FABQ: Fear-Avoidance Beliefs Questionnaire, TSK: Tampa Scale of Kinesiophobia, QuickDASH: Quick Disability Arm Shoulder Hand questionnaire. Variables are expressed as mean ± standard deviation. ** *p* < 0.001, * *p* < 0.05.

**Table 3:** Correlations between physical factors, pronociceptive pain variables, psychological measures and disability.

		Disability	Psychological Vulnerability				Physical Factors			
		QuickDASH	FABQ1	FABQ2	FABQ Total	TSK	VAS Right	VAS Left	Dynamometry Right	Dynamometry Left
Pain intensity										
Physical factors	VAS right	0.779 **	0.674 **	0.182	0.455 **	0.440 *	1	0.346	−0.480 **	−0.510 **
	VAS left	0.396 *	0.211	0.462 *	0.427 *	0.194	0.346	1	−0.176	−0.225
	Dynamometry right	−0.403 *	−0.403 **	−0.147	−0.286	−0.420 *	−0.480 **	−0.176	1	0.957 **
	Dynamometry left	−0.444 **	−0.330	−0.179	−0.272	−0.364 *	−0.510 **	−0.225	0.957 **	1
Pronociceptive pain variables	Shoulder algometry right	−0.770 **	−0.729 **	−0.397 **	−0.626 **	−0.555 **	−0.658 **	−0.452 *	0.450 **	0.456 **
	Shoulder algometry left	−0.759 **	−0.630 **	−0.353 **	0.544 **	−0.481 **	−0.680 **	−0.491 **	0.602 **	0.643 **
	Trapezium algometry right	−0.659 **	−0.543 **	−0.270	−0.438 **	−0.236	−0.540 **	−0.412 *	0.477 **	0.461 **
	Trapezium algometry left	−0.668 **	−0.552 **	−0.287	−0.454 **	−0.268	−0.667 **	−0.553 **	0.518 **	0.542 **
Disability	QuickDASH	1	0.781 **	0.406 *	0.657 **	0.414 *	0.779 **	0.369 **	−0.403 *	−0.444 **

QuickDASH: Quick Disability Arm Shoulder Hand questionnaire, VAS: visual analogue scale, TSK: Tampa Scale of Kinesiophobia, FABQ: Fear-Avoidance Beliefs Questionnaire. * *p* < 0.05; ** *p*< 0.001.

## Data Availability

The data presented in this study are available on request from the corresponding author (lauralopez@ugr.es).
